# Supplementary material for: sTREM2 cerebrospinal fluid levels are a potential biomarker for microglia activity in early‐stage Alzheimer's disease and associate with neuronal injury markers
Source: EMBO Mol Med. 2016 Mar 3;8(5):466–76. doi: 10.15252/emmm.201506123 (PMC5120370; doi:10.15252/emmm.201506123)
Supplement: Supplementary file 1 — Appendix [file EMMM-8-466-s001.pdf]

APPENDIX

Content

APPENDIX SUPPLEMENTARY METHODS 2
Control group criteria for each center 2
sTREM2 ELISA protocol 3
APPENDIX TABLES 4
Appendix Table S1. CSF core biomarkers cut-offs for each center. 4
Appendix Table S2. Summary of the linear mixed model analysis in subjects with an age ≥65yo 5
Appendix Table S3. Demographic and clinical data for the total study population. 6
Appendix Table S4. CSF sTREM2 values and number of subjects studied per center and diagnostic group. 7
APPENDIX REFERENCES 8

APPENDIX SUPPLEMENTARY METHODS

Control group criteria for each center

I. Gothenburg, Sweden
Controls subjects were cognitively normal individuals with a normal cognition (CDR=0), no history of neurological diseases and all three AD CSF core biomarkers were normal.

II. Bonn, Germany
Control subjects were recruited from patients undergoing routine lumbar punctures during neurological work up for diseases other than neurodegenerative or neuroinflammatory diseases or acute CNS injury by vascular or other causes. Neurological/psychiatric diagnoses reached were unspecified visual disturbance (n=1), non-inflammatory polyneuropathy (n=3), depression (n=7) and somatoform disorder (n=1). Controls had no record of subjective or objective cognitive impairment, and all three AD CSF core biomarkers were normal.

III. Antwerp, Belgium
The control group consisted of cognitively healthy elderly in whom cognitive deterioration was ruled out by means of neuropsychological screening. In addition to a nondisease control (n=1), the control group contained patients with the following neurological diseases: polyneuropathy (n=4), headache (n=2), atypical pain syndrome (n=1) and peripheral cranial nerve paralysis (n=3). In all these patients neurodegenerative disorders were ruled out by means of an extensive neurological work-up (Struyfs *et al*, 2015). All three AD CSF core biomarkers were normal.

IV. Clinic Barcelona, Spain
Control subjects were cognitively normal people defined according to the following criteria: Mini Mental State Examination (MMSE) scores above 24, objective cognitive performance within the normal range (performance within 1.5 SD) in all tests from a specific test battery, clinical dementia rating (CDR) scale score of 0, no significant psychiatric symptoms or previous neurological disease and all three AD CSF core biomarkers were normal.

V. Sant Pau Barcelona, Spain
Control subjects were recruited among patients' caregivers. All underwent formal cognitive evaluation using a previously published neuropsychological battery (Sala *et al*, 2008). Control subjects did not have cognitive complaints and the results of the neuropsychological evaluation were in the normal range for age and education (Alcolea *et al*, 2014). All three AD CSF core biomarkers were normal.

sTREM2 ELISA protocol

The sTREM2 ELISA was previously established by our group using the Mesoscale platform (Kleinberger *et al*, 2014). In brief, it consists of a biotinylated polyclonal goat anti-human TREM2 antibody (R&D Systems BAF1828) as capture antibody; a monoclonal mouse anti-human TREM2 antibody (Santa Cruz Biotechnology; B-3, sc373828) as a detection antibody; and a SULFO-TAG–labeled anti-mouse secondary antibody (Meso Scale Discovery). Recombinant human TREM2 protein (Hölzel Diagnostika) was used as a standard (4000 to 62.5 pg/ml).

Streptavidin-coated 96-well plates were blocked overnight at 4°C in blocking buffer [0.5% bovine serum albumin (BSA) and 0.05% Tween 20 in PBS (pH 7.4)]. The plates were next incubated with the capture antibody diluted in the blocking buffer during 1 hour at room temperature. They were subsequently washed four times with washing buffer (0.05% Tween 20 in PBS) and incubated for 2 hours at room temperature with the CSF samples diluted 1:4 in assay buffer [0.25% BSA and 0.05% Tween 20 in PBS (pH=7.4)] supplemented with protease inhibitors (Sigma) or the recombinant human TREM2 protein. Plates were again washed three times with washing buffer before incubation for 1 hour at room temperature with the detector antibody. After three additional washing steps, plates were incubated with the secondary antibody and incubated for 1 hour in the dark. Last, plates were washed three times with wash buffer followed by two washing steps in PBS. The electrochemical signal was developed by adding Meso Scale Discovery Read buffer and the light emission measured using the Mesoscale Discovery SECTOR Imager 2400.

In a preliminary study (Kleinberger *et al*, 2014), the initial spike recovery (108.3%), linearity (95.7%), interplate and interday variability (both <13%) was determined using a dedicated CSF sample. Minimal measurement variation (CV=10%) between freeze-thaw cycles (up to 7 cycles) was found.

We calculated the concentration of sTREM2 using a five-parameter logistic curve fitting method with the MasterPlex ReaderFit software (MiraiBio Group, Hitachi Solutions America).

APPENDIX TABLES

Appendix Table S1. CSF core biomarkers cut-offs for each center.

Center	T-tau	P-tau$_{181P}$	Aβ_{1-42}
**I. Gothenburg, Sweden**	400	60/80*	550
**II. Bonn, Germany**	450	60	¶
**III. Antwerp, Belgium**	296.5	56.5	638.5
**IV. Clinic Barcelona, Spain**	†	75	495/550§
**V. Sant Pau Barcelona, Spain**	350	61	550

All values are in pg/mL.
* P-tau$_{181P}$ cut-offs depending on age:
  - <60yo: 60
  - ≥60yo: 80

¶ Aβ_{1-42}/Aβ_{1-40}=0.09 (measured by MSD).
† T-tau cut-offs depending on age:
  - <50yo: 300
  - 51-70yo: 450
  - >70yo: 500

§ Depending on the sample batch.

Appendix Table S2. Summary of the linear mixed model analysis in subjects with an age ≥65yo

Diagnostic group	Unadjusted Mean	95 % CI	Adjusted Mean*	95 % CI	n
Control	-0.347	-0.398, -0.296	-0.310	-0.411, -0.209	63
Preclinical AD	-0.223	-0.308, -0.138	-0.212	-0.318, -0.107	46
MCI-AD	-0.136†	-0.180, -0.093	-0.143§¶	-0.240, -0.046	101
AD dementia	-0.195‡	-0.241, -0.149	-0.224	-0.320, -0.127	159

CSF sTREM2 levels are expressed in their log-transformed values. They are shown as unadjusted means and 95% CI (p values calculated by one-way ANOVA), and adjusted (*) for gender and age (fixed effects) and center (random effects) in a linear mixed model.
Abbreviations: AD, Alzheimer's disease; CI, confidence interval; CSF, cerebrospinal fluid; MCI-AD, MCI due to AD.
Adjustments based on age mean = 76.07. Post-hoc comparisons (Bonferroni):
† $p<0.0001$ vs control
‡ $p=0.001$ vs control
§ $p=0.0005$ vs control
¶ $p=0.066$ vs dementia AD
Note that, in subjects that are 65 years or older, CSF sTREM2 is significantly increased in prodromal AD compared to the control group, and there is a tendency to be increased compared to the dementia AD group.

Appendix Table S3. Demographic and clinical data for the total study population.

Variable	n	Females, %	APOE ε4 carriers, %	Age, years		CSF biomarkers				CSF sTREM2	
**Diagnostic group**						$A\beta_{1\text{-}42}$	T-tau	P-tau$_{181P}$		Relative to an IS	ng/mL
**Controls**	150	59%	21%	62.4 (11)		796 (159)	218 (81)	43 (12)		0.470 (0.202)	3.07 (1.4)
**AD continuum subjects**											
Preclinical AD	63	60%	58%	70.8 (11)		414 (98)	450 (428)	66 (39)		0.644 (0.41)	4.09 (2.7)
MCI-AD	111	60%	52%	74.3 (9)		426 (107)	737 (410)	95 (32)		0.802 (0.38)	5.98 (3.2)
AD dementia	200	62%	62%	73.8 (10)		408 (113)	920 (564)	102 (44)		0.725 (0.44)	5.33 (3.7)
**Other groups**											
SNAP	39	49%	26%	68.2 (10)		990 (289)	466 (228)	78 (34)		0.751 (0.34)	5.44 (2.6)
MCI-noAD	103	45%	32%	69.3 (9)		667 (261)	305 (199)	48 (18)		0.489 (0.23)	3.22 (1.6)

Data are expressed as percent (%) or mean (SD), as appropriate. Abbreviations: Aβ, amyloid β-peptide; AD, Alzheimer disease; APOE, apolipoprotein E; CSF, cerebrospinal fluid; F, Female; IS, internal standard; M, Male; MCI, mild cognitive impairment; P-tau$_{181P}$, tau phosphorylated at threonine 181; SNAPs: suspected non-AD pathology; T-tau, total tau.
APOE genotype only available in 103 controls (69%), 39 Preclinical AD (62%), 89 MCI-AD (80%), 148 AD Dementia (74%), 27 (69%) SNAP and in 73 (71%) MCI-noAD. Only $A\beta_{1\text{-}42}$ measured by the Innotest ELISA are included; $A\beta_{1\text{-}42}$ values from Bonn group (measured by MSD) are excluded.

Appendix Table S4. CSF sTREM2 values and number of subjects studied per center and diagnostic group.

	I	II	III	IV	V	
**Diagnostic group**	**Gothenburg, Sweden**	**Bonn, Germany**	**Antwerp, Belgium**	**Clinic Barcelona, Spain**	**Sant Pau Barcelona, Spain**	**TOTAL**
**Controls**	0.548 (0.20) [24]	0.452 (0.18) [18]	0.556 (0.16) [11]	0.353 (0.11) [31]	0.489 (23) [66]	150
**AD continuum subjects**						
Preclinical AD	0.921 (0.37) [14]	1.020 (0.40) [5]	0.775 (0.41) [7]	0.499 (0.34) [23]	0.406 (0.33) [14]	63
MCI-AD	1.041 (0.40) [6]	0.800 (0.42) [16]	0.968 (0.36) [40]	0.725 (0.32) [25]	0.547 (0.25) [24]	111
AD dementia	0.851 (0.35) [22]	0.754 (0.44) [52]	0.871 (0.50) [65]	0.617 (0.32) [20]	0.440 (0.25) [41]	200
**Other groups**						
SNAP	NA [0]	0.774 (0.36) [6]	0.749 (0.30) [13]	0.849 (0.36) [9]	0.659 (0.37) [11]	39
MCI-noAD	0.648 (0.17) [10]	0.512 (0.26) [36]	NA [0]	0.396 (0.18) [12]	0.460 (0.21) [45]	103
**TOTAL**	76	133	136	120	201	666

The table contains the mean (SD) of CSF sTREM2 (relative to an internal standard) and the number of subjects (in square brackets) included per group and center. Abbreviations: AD, Alzheimer disease; CSF, cerebrospinal fluid; MCI, mild cognitive impairment; SNAPs: suspected non-AD pathology; NA, not applicable; SNAPs: suspected non-AD pathology.

APPENDIX REFERENCES

Alcolea D, Carmona-Iragui M, Suárez-Calvet M, Sánchez-Saudinós MB, Sala I, Antón-Aguirre S, Blesa R, Clarimón J, Fortea J, Lleó A (2014) Relationship between β-secretase, inflammation and core cerebrospinal fluid biomarkers for Alzheimer's disease. *J Alzheimers Dis* 42**:** 157–167

Kleinberger G, Yamanishi Y, Suárez-Calvet M, Czirr E, Lohmann E, Cuyvers E, Struyfs H, Pettkus N, Wenninger-Weinzierl A, Mazaheri F, Tahirovic S, Lleó A, Alcolea D, Fortea J, Willem M, Lammich S, Molinuevo JL, Sánchez-Valle R, Antonell A, Ramirez A, et al (2014) TREM2 mutations implicated in neurodegeneration impair cell surface transport and phagocytosis. *Sci Transl Med* 6**:** 243ra86

Sala I, Belén Sánchez-Saudinós M, Molina-Porcel L, Lázaro E, Gich I, Clarimón J, Blanco-Vaca F, Blesa R, Gómez-Isla T, Lleó A (2008) Homocysteine and cognitive impairment. Relation with diagnosis and neuropsychological performance. *Dement Geriatr Cogn Disord* 26**:** 506–12

Struyfs H, Van Broeck B, Timmers M, Fransen E, Sleegers K, Van Broeckhoven C, De Deyn PP, Streffer JR, Mercken M, Engelborghs S (2015) Diagnostic Accuracy of Cerebrospinal Fluid Amyloid-β Isoforms for Early and Differential Dementia Diagnosis. *J Alzheimers Dis* 45**:** 813–822
